# High-performance elastic ferroelectrics *via* low-temperature carbene crosslinking and high-temperature annealing†

**DOI:** 10.1039/d5sc01467k

**Published:** 2025-04-29

**Authors:** Linping Wang, Liang Gao, Xiaocui Rao, Fangzhou Li, Da Zu, Yunya Liu, Ben-Lin Hu

**Affiliations:** a Advanced Interdisciplinary Sciences Research (AiR) Center, Ningbo Institute of Materials Technology and Engineering, Chinese Academy of Sciences 1219 West Zhongguan Road, Zhenhai District Ningbo 315201 P. R. China hubenlin@nimte.ac.cn; b College of Materials Science and Opto-Electronic Technology, University of Chinese Academy of Sciences No. 1 Yanqihu East Rd, Huairou District Beijing 101408 P. R. China; c Ordered Matter Science Research Center, Nanchang University 339 Beijing East Road, Qingshanhu District Nanchang Jiangxi Province 330029 P. R. China; d Key Laboratory of Low Dimensional Materials and Application Technology, Ministry of Education, School of Materials Science and Engineering, Xiangtan University Yuhu District Xiangtan Hunan 411105 P. R. China

## Abstract

With the increasing demand for wearable electronics, elastic ferroelectrics with high polarization intensity and Curie temperature have become essential. However, balancing high ferroelectric performance with elasticity in polymeric ferroelectrics remains a challenge, as higher crosslinking density to improve elasticity often compromises Curie temperature and remnant polarization. To address this trade-off, we introduce unsaturated bonds into commercial P(VDF-TrFE), forming P(VDF-TrFE-DB) with enhanced crosslinking reactivity while retaining its inherent ferroelectric properties. A novel two-step LT–HT processing strategy is developed to achieve this balance. The low-temperature (LT) step leverages carbene-mediated crosslinking with diazirine-based crosslinkers below the polymer's Curie temperature, preventing premature crystallization and forming amorphous regions essential for mechanical flexibility. The high-temperature (HT) annealing step promotes the formation and alignment of well-ordered ferroelectric crystalline structures, optimizing remnant polarization and Curie temperature while preserving the crosslinked amorphous regions critical for elasticity. This approach enables high elasticity with minimal crosslinker content while maintaining excellent ferroelectric performance. The resulting elastic P(VDF-TrFE-DB) polymer exhibits a significantly elevated Curie temperature (∼140 °C) and high remnant polarization (7.63 μC cm^−2^), comparable to commercial P(VDF-TrFE). This method offers a versatile pathway for advanced flexible electronics, soft actuators, and wearable devices requiring robust mechanical and ferroelectric properties.

## Abstract 2

Novel elastic ferroelectrics, developed by introducing DBs into commercial P(VDF-TrFE) and applying an innovative two-step LT–HT process, show high *T*_c_ and *P*_r_ with excellent recovery, setting a new benchmark for flexible electronic materials.

## Graphical abstract

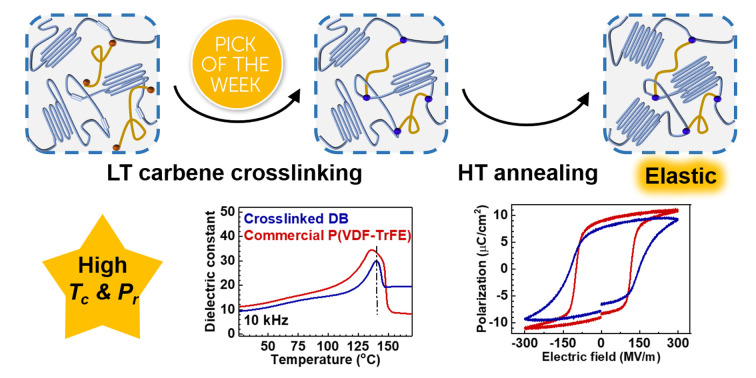

## Introduction

Elastic or flexible ferroelectrics are emerging as pivotal materials for wearable and implantable technologies,^1,2^ offering unique capabilities for actuators, energy harvesters, and nonvolatile memories.^3,4^ Their versatility arises from properties such as switchable spontaneous polarization, high dielectric permittivity, and electromechanical coupling performance.^5–8^ Considerable efforts have focused on developing novel ferroelectrics that combine mechanical elasticity with high ferroelectric performance,^9–11^ including enhanced longitudinal piezoelectric coefficients (*d*_33_),^12^ high dielectric constants,^13,14^ elevated Curie temperatures (*T*_c_),^15^ and strong polarization. Importantly, different applications demand distinct properties. For instance, relaxor ferroelectric polymers typically exhibit high dielectric constants, low Curie temperatures, and slim *P*–*E* hysteresis loops, making them ideal for energy storage and actuator applications.^16–18^ Conversely, traditional ferroelectrics with high *T*_c_ and strong polarization, exhibiting square *P*–*E* loops, are ideal for non-volatile memory devices.^19,20^ This diversity necessitates the tailored development of ferroelectric materials to meet application-specific demands.

Recently, flexible ferroelectrics with a high *d*_33_ value have been developed as implantable piezoelectric materials,^12^ demonstrating immense potential as mechanical-electrical transducers for biomedical treatments. These materials were precisely designed using the strategy of molecular ferroelectric chemistry.^21,22^ Additionally, elastic ferroelectrics with high dielectric constants and low dielectric losses have been achieved by crosslinking relaxor ferroelectric polymers with long- or short-chain crosslinkers.^13,14^ These materials exhibit significantly higher dielectric constants compared to pristine relaxor ferroelectric polymers. However, elastic ferroelectric polymers of the normal type still face limitations, particularly in terms of their *T*_c_ and polarization values,^9,15^ which remain significantly lower than those of commercial P(VDF-TrFE) 80/20 mol%, widely regarded as having optimal ferroelectric properties among P(VDF-TrFE) copolymers.^23,24^ A critical challenge lies in achieving elasticity, without compromising crystallinity, which is essential for achieving high *T*_c_ and remnant polarization (*P*_r_).

Existing approaches to preparing normal elastic ferroelectrics often sacrifice crystallinity to enhance elasticity. For example, commercial P(VDF-TrFE) 55/45 mol% offers improved stretchability but reduced crystallinity,^9^ while modifications of P(VDF-CTFE) 80/20 mol% to create P(VDF-TrFE-DB) (“old DB”) yield relatively higher *T*_c_ but still fall short of the ferroelectric properties of commercial P(VDF-TrFE) 80/20 mol%.^15^ Despite the high VDF content of old DB, the material exhibited a *T*_c_ of only ∼85 °C, limited by regio-defects such as “head-to-head” structures.^25^ These findings underscore the ongoing trade-off between elasticity and ferroelectricity in elastic ferroelectrics, necessitating innovative strategies to overcome these challenges.

To address these challenges, we strategically introduced unsaturated C=C bonds directly into the polymer backbone of commercially available P(VDF-TrFE) 80/20 mol%, resulting in a novel polymer, P(VDF-TrFE-DB) (“new DB”), which combines enhanced stretchability with a comparable Curie temperature. These unsaturated bonds not only improve the material's stretchability but also increase its crosslinking reactivity. Furthermore, we employed a two-step LT–HT process consisting of low-temperature crosslinking followed by high-temperature annealing to optimize material properties. The LT step is performed at a temperature below the Curie temperature of P(VDF-TrFE-DB), ensuring that crosslinking occurs without initiating crystallization into large domains. This controlled low-temperature (LT) crosslinking promotes the formation of a network structure in the amorphous regions, which enhances material plasticization and elasticity while minimizing the use of crosslinkers. The subsequent high-temperature (HT) annealing step facilitates the formation of functional crystalline domains,^26^ preserving the material's high ferroelectric performance by promoting the alignment of dipoles within the crystalline regions. This LT–HT process effectively balances elasticity and ferroelectricity by selectively controlling both the amorphous and crystalline phases. The carbene-based crosslinkers used in this process, known for their high reactivity,^27–29^ ensure efficient crosslinking with minimal impact on the material's ferroelectric properties, thus enhancing both elasticity and ferroelectricity.

By leveraging the high-Curie-temperature ferroelectric polymer P(VDF-TrFE-DB) in conjunction with the LT–HT thermal process, we successfully developed intrinsically elastic ferroelectrics with a high *T*_c_ and *P*_r_ comparable to those of commercial P(VDF-TrFE) 80/20 mol%. This innovative strategy offers a promising pathway to reconcile the trade-off between elasticity and ferroelectricity in polymer-based materials.

## Results and discussion

### Strategic design and preparation of the elastic polymer with high *T*_c_ and *P*_r_

Developing elastic ferroelectric materials that simultaneously achieve high ferroelectric performance and elasticity requires a balance between high crystallinity and plasticity. High crystallinity is essential for achieving a high *T*_c_ and high polarization, while sufficient plasticity enables recoverable network formation. Among β-phase polymers, commercial P(VDF-TrFE) 80/20 mol% stands out because of its high *T*_c_ and large *P*_r_.^30^ However, its tensile break strain of less than 10% after annealing makes it unsuitable as a pristine polymer for elastic ferroelectrics.^9^

**Fig. 1:**
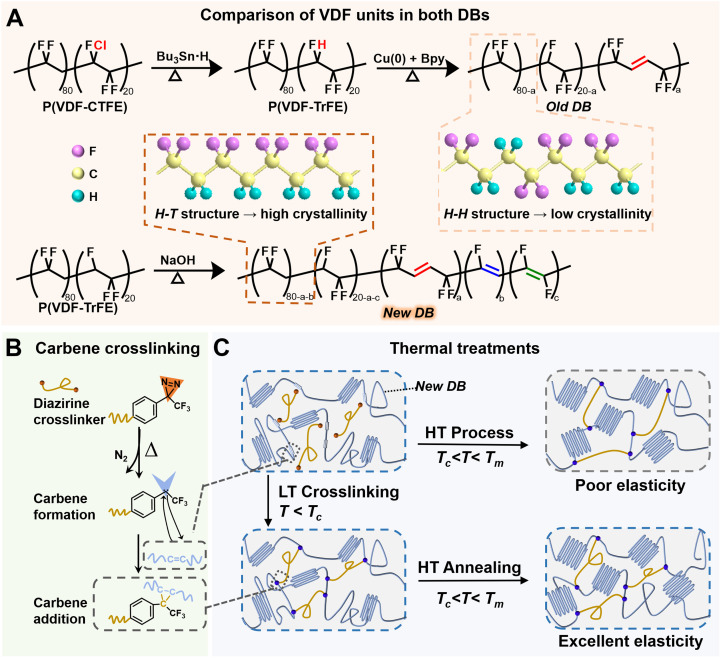
Strategic design of an elastic ferroelectric with enhanced Curie temperature and polarization. (A) Schematic comparison between the novel stretchable ferroelectric polymer (new DB) introduced in this work and a previously used polymer (old DB), highlighting the structural improvements that promote a highly crystalline configuration. (B) Mechanism of the low-temperature carbene-mediated crosslinking process. (C) Diagram comparing the LT–HT process with the conventional one-step HT process, highlighting the advantages of sequential low-temperature plasticization and high-temperature crystalline development.

To overcome this limitation, we recently developed a method to enhance the stretchability of commercial P(VDF-TrFE) by introducing double bonds (DB) *via* alkaline treatment.^31^ Based on this approach, we modified the method to incorporate DB directly into commercial P(VDF-TrFE) 80/20 mol%, creating P(VDF-TrFE-DB) (referred to as “new DB”), as depicted in Fig. 1A. Compared to the “old DB” derived from P(VDF-CTFE), the new DB, although containing three types of double bonds, is derived from P(VDF-TrFE) with a high head-to-tail (H–T) configuration, which promotes greater crystallinity and consequently leads to a higher *T*_c_ and *P*_r_. Moreover, the introduction of DB enhances both the stretchability and chemical reactivity of the polymer. Carbene crosslinking, specifically, carbene addition (Fig. 1B),^15,32^ is a chemical reaction in which a carbene, a highly reactive species containing a neutral carbon atom with two non-bonded electrons, can add to the π-bond of the new DB, thereby forming cyclopropane rings as crosslinking points. This reaction allows effective crosslinking even under mild conditions, such as low temperature.

In our prior work, PEG2000-diazirine was employed as a crosslinker for carbene crosslinking, with a crosslinker-to-polymer ratio of 6 : 10 proving effective for plasticization but adversely impacting polarization.^15^ To address this limitation, a two-step process, low-temperature crosslinking followed by high-temperature annealing (LT–HT), was employed in this work. Fig. 1C illustrates the key structural differences between the conventional one-step HT process and the LT–HT approach. The HT process results in uncontrolled crystallization, yielding materials with a reduced amorphous region and compromised elasticity. Conversely, the LT–HT process enables a controlled structure comprising crosslinked amorphous regions alongside well-formed crystalline domains, delivering exceptional mechanical resilience and ferroelectricity.

In detail, the LT step is conducted at a temperature below the *T*_c_ of P(VDF-TrFE-DB) to prevent premature crystallization, which typically leads to the formation of large, uncontrolled crystalline domains that impede crosslinking. During this step, carbene crosslinkers react with unsaturated bonds in the polymer backbone, forming chemical crosslinks within the amorphous regions. The incorporated PEG chains from crosslinkers plasticize the amorphous matrix,^33^ enhancing flexibility while maintaining a network structure vital for mechanical resilience. Subsequently, the HT annealing step facilitates the formation and growth of well-ordered ferroelectric crystalline domains, ensuring optimal packing and alignment of polymer chains to maximize *T*_c_ and *P*_r_.^34^ Crucially, the crosslinked network established in the amorphous regions remains intact, preserving mechanical integrity and reversible elastic deformation under cyclic loading. This two-step LT–HT process strategically balances the structural roles of amorphous and crystalline regions, allowing for the integration of elasticity and robust ferroelectricity, enabling the material to withstand mechanical deformation without compromising its ferroelectric properties.

To substantiate the feasibility of this LT–HT process, extensive characterization studies were performed. The ^1^H NMR spectra of new DB (Fig. S1†) confirmed the successful incorporation of DB, with characteristic signals at 6.1–7.0 ppm after alkaline treatment. The calculated double content is 1.8 mol%. ^19^F NMR analysis (Fig. S2, Table S1 and S2†) indicated that commercial P(VDF-TrFE) 80/20 mol% contains a higher proportion of H-T configurations and fewer regiodefects.^35–37^ These structural advantages result in significantly higher *T*_c_ (136 °C), melting temperature (*T*_m_, 146 °C), and enthalpy values (54 J g^−1^) for new DB (Fig. S3 and Table S3†). Based on these findings, the LT process should be conducted at a temperature below 136 °C, while the subsequent HT annealing should be performed between 136 °C and 146 °C.

**Fig. 2:**
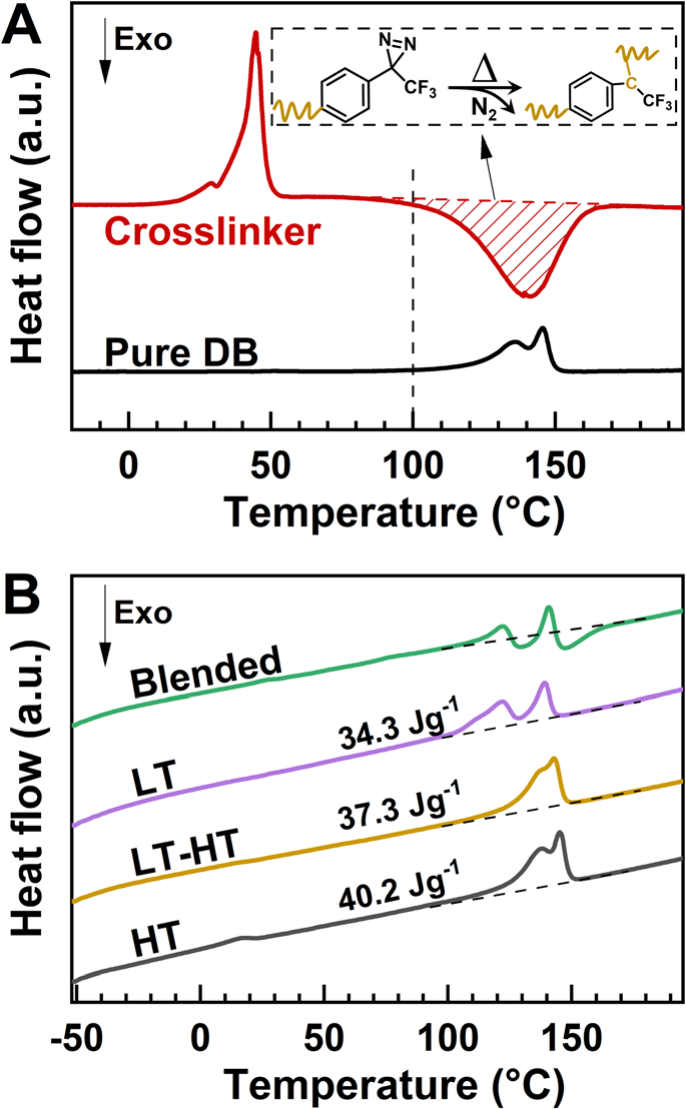
Evaluation of crosslinking efficiency in LT and LT–HT processes. (A) DSC curves of pure DB and the PEG-diazirine crosslinker during the first heating cycle, with the red-shaded region highlighting the crosslinking reaction and the inset chart showing the crosslinking reaction. (B) DSC curves of the blended sample (dried at 60 °C), LT-processed sample, LT–HT-processed sample, and HT-processed sample during the first heating cycle.

As shown in Fig. 2A, PEG2000-diazirine exhibits reactivity starting around 80 °C, with a distinct PEG melting peak at 40 °C. Based on these thermal characteristics, 100 °C was chosen for the LT crosslinking step, which was carried out over 24 hours to ensure complete crosslinking while suppressing additional crystallinity development. Subsequently, annealing at 140 °C, just below a *T*_m_ of 146 °C, promoted the formation of large crystalline domains while maintaining the crosslinked amorphous regions critical for elasticity. The effectiveness of crosslinking in the LT process was validated using differential scanning calorimetry (DSC) analysis, with a sample at the desired crosslinking density of 1.5% serving as an example (Fig. 2B). Samples dried at 60 °C exhibited an exothermic peak, indicative of active crosslinking reactions, while LT-processed samples showed no such peak, confirming the completion of crosslinking. Furthermore, compared to LT- or HT-processed samples, the LT–HT sample exhibited a moderate enthalpy value of 37.3 J g^−1^, supporting the hypothesis that post-crosslinking annealing maintains a balanced structural configuration. The crosslinking density, defined in detail in Table S4,† was fine-tuned by varying the crosslinker ratio to strike an optimal balance between resilience and VDF crystallinity.

### Mechanical properties of crosslinked samples *via* different heating processes

**Fig. 3:**
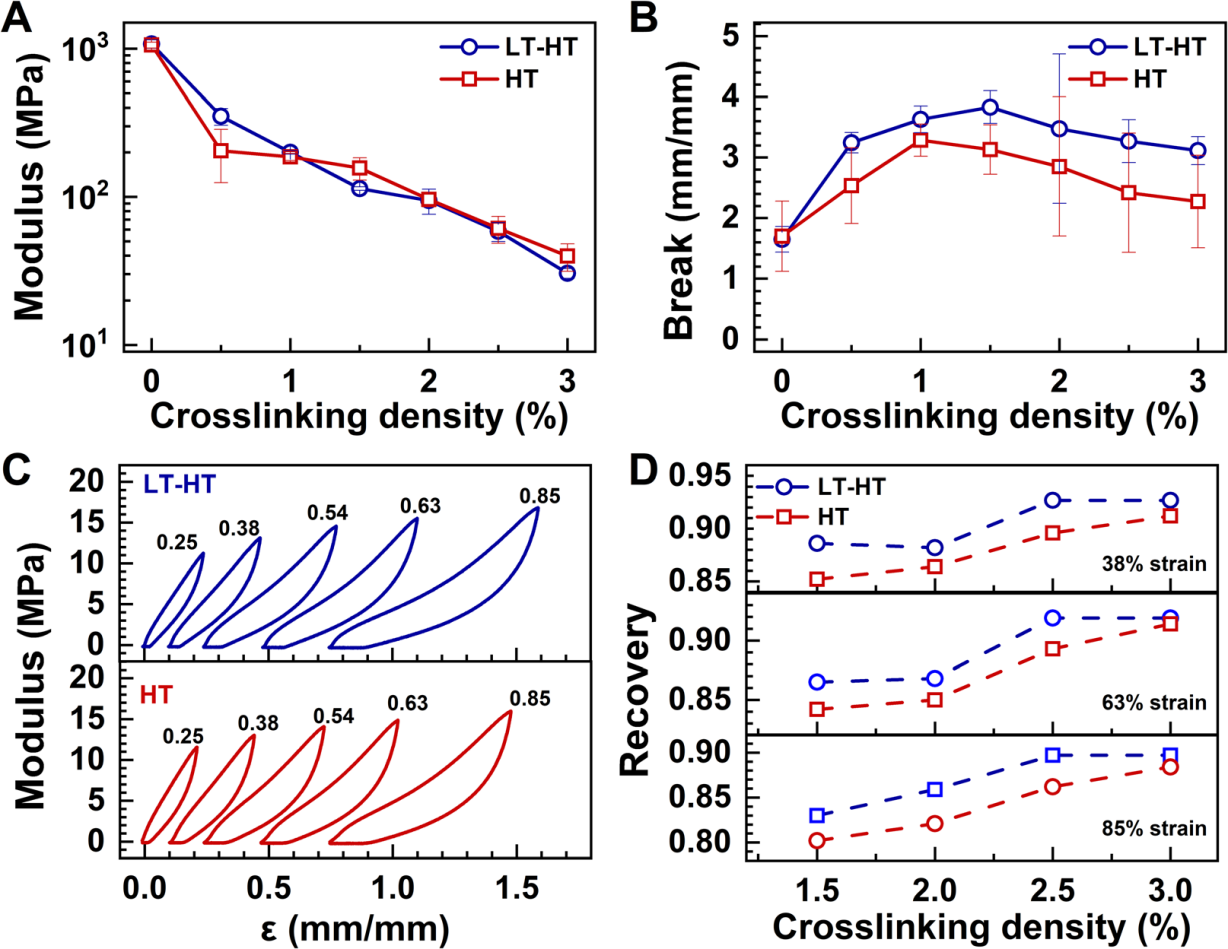
Mechanical properties of samples with varying crosslinking densities and thermal treatments. (A) Young's modulus and (B) elongation at break for samples with varying crosslinking densities under two thermal treatments. (C) Elastic recovery curves comparing LT–HT-processed and HT-processed samples with 1.5% crosslinking density under varying strains. (D) Resilience of samples with varying crosslinking densities under different strains, showing higher elastic recovery in LT–HT-processed samples across all crosslinking densities.

The differences in crystallinity resulting from the LT–HT and HT heating processes significantly impact the mechanical properties of the crosslinked samples. While both LT–HT-processed and HT-processed samples exhibit comparable Young's modulus values (Fig. 3A, S4 and S5†), LT–HT-processed samples demonstrate superior tensile break values, reflecting enhanced stretchability. At a crosslinking density of 1.5%, both LT–HT-processed and HT-processed samples exhibited peak tensile break values (Fig. 3B). This trend may be governed by a balance between two opposing effects: the plasticizing influence of the PEG chains within the P(VDF-TrFE-DB) matrix,^38,39^ which enhances flexibility, and the rigidity imparted by increased crosslinking density, which restricts deformation. The balance between these opposing effects results in a maximum tensile break value at the optimal crosslinking density of 1.5%.

Elastic recovery analysis further underscores the advantages of the LT–HT process. LT–HT-processed samples exhibit slimmer hysteresis loops and higher recovery rates under identical strain conditions compared to HT-processed counterparts (Fig. 3C), indicating superior elasticity. Notably, resilience assessments across varying crosslinking densities and strain levels consistently demonstrate higher recovery values for LT–HT-processed samples (Fig. 3D). This enhanced performance can be attributed to the LT–HT process's ability to preserve amorphous regions while fostering high crystallinity. Furthermore, the durability of crosslinked P(VDF-TrFE-DB) with a crosslinking density of 1.5%, prepared using the LT–HT process, was evaluated through cyclic stress–strain measurements under 50% strain. Remarkably, the material maintained excellent recovery over extended cycles, exceeding 80% recovery during the initial 100 cycles and remaining stable beyond 500 cycles of stretching and releasing (Fig. S6†). Even after 7000 cycles, the elastic recovery did not exhibit significant degradation, outperforming previously reported crosslinked DBs. This elasticity arises primarily from entropy elasticity rather than energy elasticity, as evidenced by the “force–temperature relationships” of crosslinked P(VDF-TrFE-DB) under different strains (Fig. S7†).

These findings highlight the superiority of the LT–HT process in achieving optimal mechanical properties for crosslinked ferroelectric polymers. By preserving amorphous regions and promoting high crystallinity, LT–HT processing enables a unique combination of tensile strength, elasticity, and durability.

### Crystalline properties of elastic ferroelectric films

**Fig. 4:**
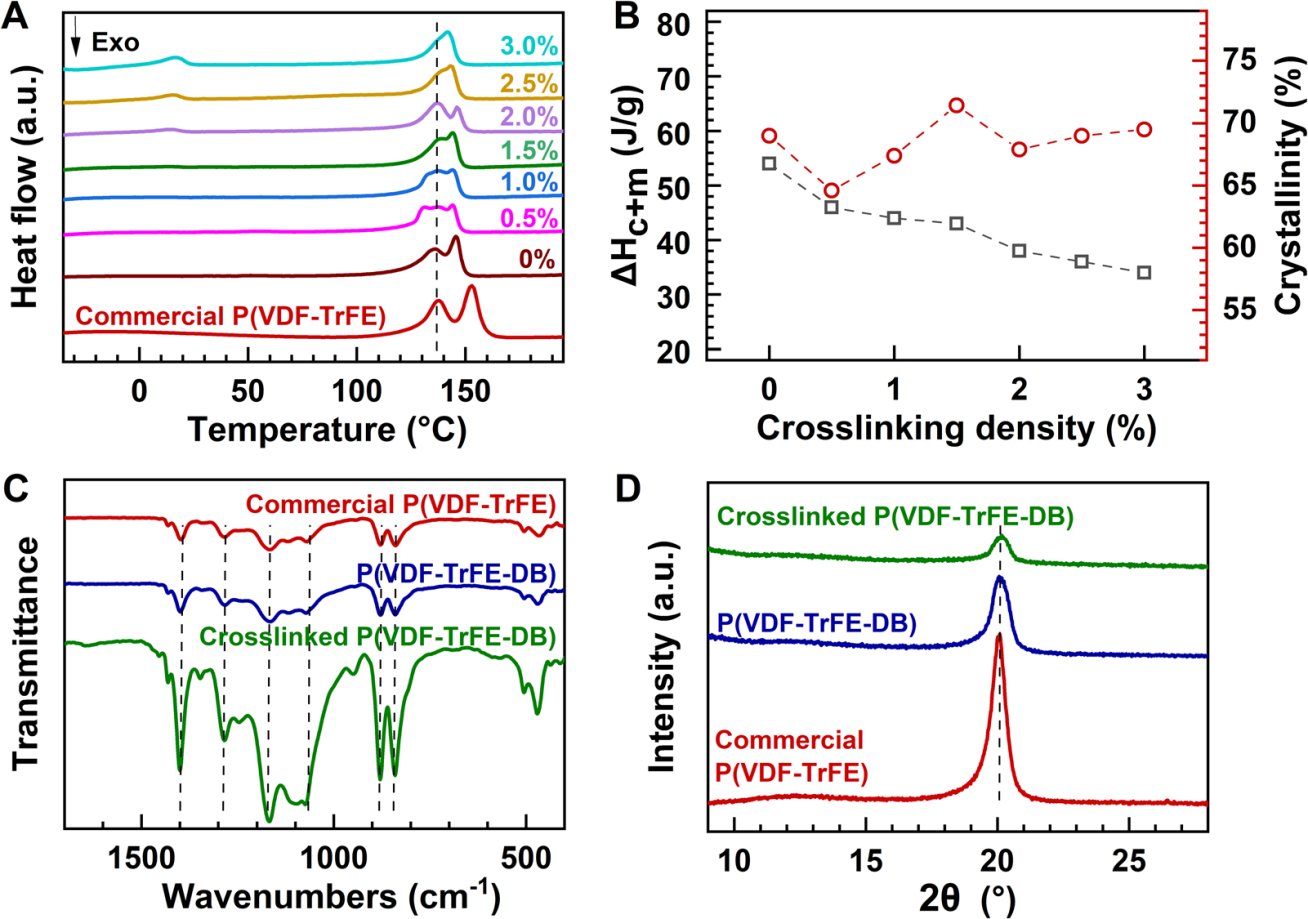
Crystalline properties of different samples. (A) DSC curves, (B) enthalpy and VDF crystallinity of the crosslinked DBs at varying crosslinking densities during the first heating cycle; (C) FTIR spectra and (D) XRD patterns of the commercial P(VDF-TrFE) 80/20 mol%, pristine P(VDF-TrFE-DB), and crosslinked samples, with dashed lines indicating the characteristic β-phase peaks.

Since LT–HT-processed crosslinked samples demonstrated superior elasticity, their crystalline properties were analyzed alongside those of pristine samples using DSC, FTIR, and XRD, as illustrated in Fig. 4. Both crosslinked and pristine P(VDF-TrFE-DB) demonstrated similar *T*_c_ and *T*_m_, approximately 136 °C and 146 °C, respectively (Fig. 4A). Notably, while the *T*_m_ of the crosslinked samples was lower than that of commercial P(VDF-TrFE) 80/20 mol%, the *T*_c_ remained comparable, suggesting that the ferroelectric behavior of the crosslinked samples closely matches that of the commercial material.

As the crosslinking density increased, the proportion of PEG chains in the samples also rose, leading to evident phase separation observed through optical microscopy and AFM (Fig. S8†) and a decrease in the enthalpy of the VDF units (Fig. 4B). Interestingly, the crystallinity of the VDF component peaked at a crosslinking density of 1.5% (Fig. 4B and Table S5†), potentially due to the enhancement of VDF unit crystallization by the presence of PEG crystals. Based on these findings, the LT–HT-processed crosslinked P(VDF-TrFE-DB) at 1.5% crosslinking density was identified as the optimal sample for further investigation. Thermal stability analysis revealed that the crosslinked P(VDF-TrFE-DB) films maintained an onset degradation temperature exceeding 350 °C (Fig. S9†), showcasing exceptional thermal resistance. Furthermore, the films exhibited robustness against common organic solvents such as cyclohexanone, acetone, dimethylformamide, and isophorone. Immersion tests demonstrated swelling behavior with a gel content of approximately 82%, confirming the solvent resistance of the crosslinked network (Fig. S10 and Table S6†).

To ensure the preservation of the desirable ferroelectric β-phase morphology, FTIR and XRD analyses were conducted. As shown in Fig. 4C, the FTIR spectra of crosslinked samples exhibited characteristic β-phase peaks at 840, 882, 1069, 1170, 1283 and 1401 cm^−1^,^40–42^ confirming the retention of β-phase crystallinity despite the crosslinking modifications. Additionally, XRD patterns (Fig. 4D) exhibited a prominent β-phase peak at 20.1°,^43,44^ further corroborating the structural integrity of the crystalline regions.

### Ferroelectricity of crosslinked P(VDF-TrFE-DB)

**Fig. 5:**
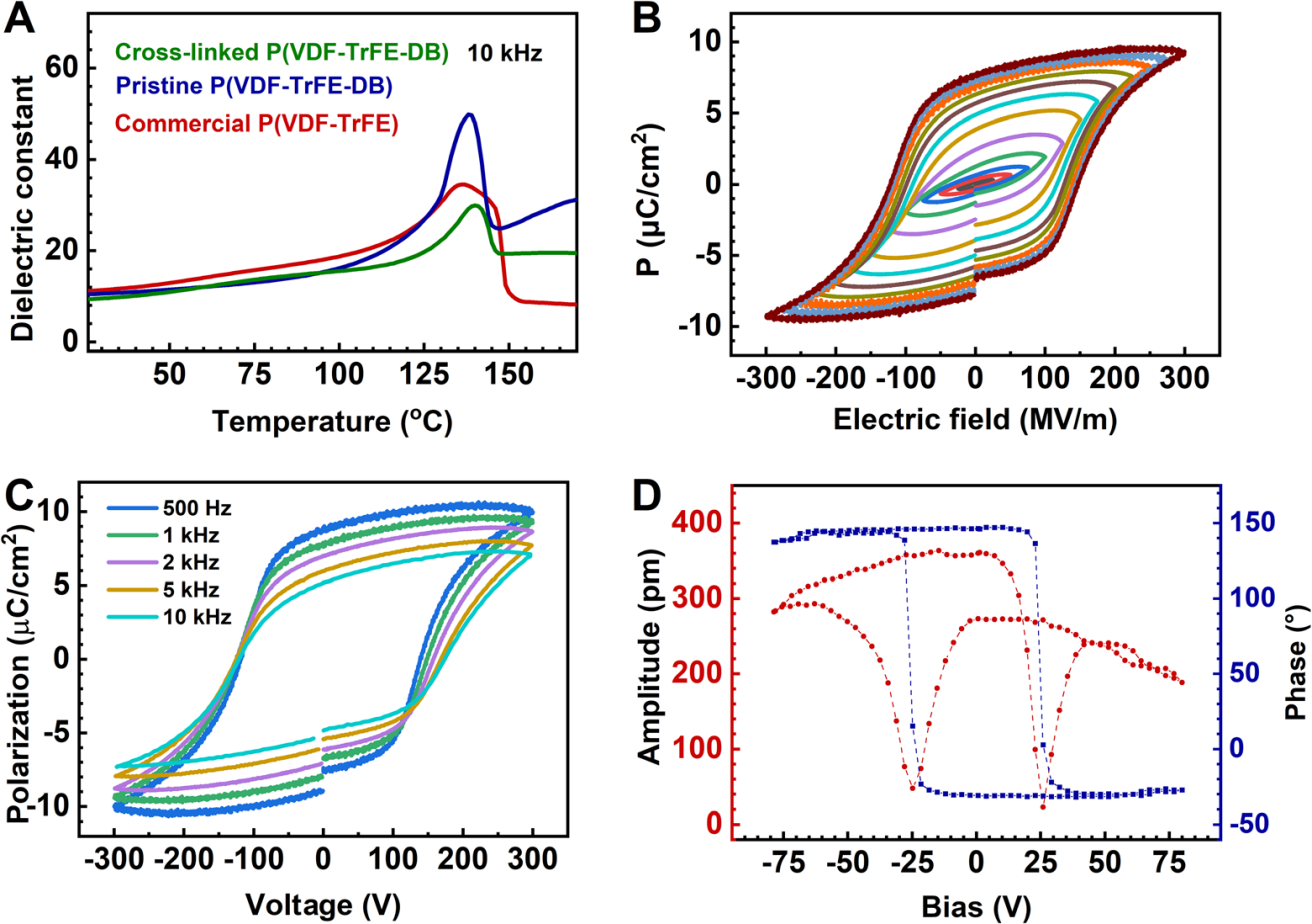
Ferroelectric properties of the crosslinked P(VDF-TrFE-DB) films. (A) *ε*–*T* curves of P(VDF-TrFE) 80/20 mol%, pristine P(VDF-TrFE-DB) and crosslinked P(VDF-TrFE-DB) films at 10 kHz. (B and C) *P*–*E* loops of Au/crosslinked P(VDF-TrFE-DB)/Au under different electric fields at 1 kHz (B) and at different frequencies (C). (D) Phase–voltage hysteresis and amplitude–voltage butterfly loop from PFM measurements.

The ferroelectric properties of crosslinked P(VDF-TrFE-DB) were characterized using temperature-dependent dielectric constant (*ε*–*T*) curves, polarization–electric field (*P*–*E*) loops, and piezoresponse force microscopy (PFM) (Fig. 5). The *ε*–*T* curves (Fig. 5A and S11†) confirmed the ferroelectric-paraelectric phase transition (“Curie transition”) in commercial P(VDF-TrFE) 80/20 mol%, pristine DB, and crosslinked P(VDF-TrFE-DB). These results demonstrated the preservation of a β-like phase across all samples. Notably, the *T*_c_ of the crosslinked polymer increased from 135 °C in commercial P(VDF-TrFE) 80/20 mol% to approximately 140 °C, consistent with the differential scanning calorimetry (DSC) data (Fig. 4A).

The *P*–*E* loop of crosslinked P(VDF-TrFE-DB) expanded with increasing electric field beyond 125 MV m^−1^, reaching saturation after 250 MV m^−1^ (Fig. 5B). The elastic ferroelectric film exhibited a coercive field (*E*_c_) of 148 MV m^−1^, higher than the *E*_c_ of 116 MV m^−1^ in the commercial P(VDF-TrFE) 80/20 mol% film (Fig. S12†). Its maximum (*P*_max_) and remanent (*P*_r_) polarizations were 9.49 μC cm^−2^ and 7.63 μC cm^−2^, respectively, compared to 11.10 μC cm^−2^ and 8.82 μC cm^−2^ for the commercial film. Despite the slightly lower polarization values, the *P*_r_ value of 7.63 μC cm^−2^ represents the highest reported among elastic ferroelectrics to date. Furthermore, frequency-dependent *P*–*E* measurements showed increasing *P*_r_ values from 5.21 μC cm^−2^ at 10 kHz to 8.68 μC cm^−2^ at 500 Hz (Fig. 5C), highlighting the frequency sensitivity of the material's ferroelectric performance. To further investigate the piezoelectric properties of the crosslinked elastic ferroelectric, PFM was employed. The phase and amplitude loops (Fig. 5D) displayed typical square and butterfly shapes,^45,46^ respectively, confirming complete ferroelectric switching in the thin film.

### Ferroelectric response of crosslinked P(VDF-TrFE-DB) under strains

**Fig. 6:**
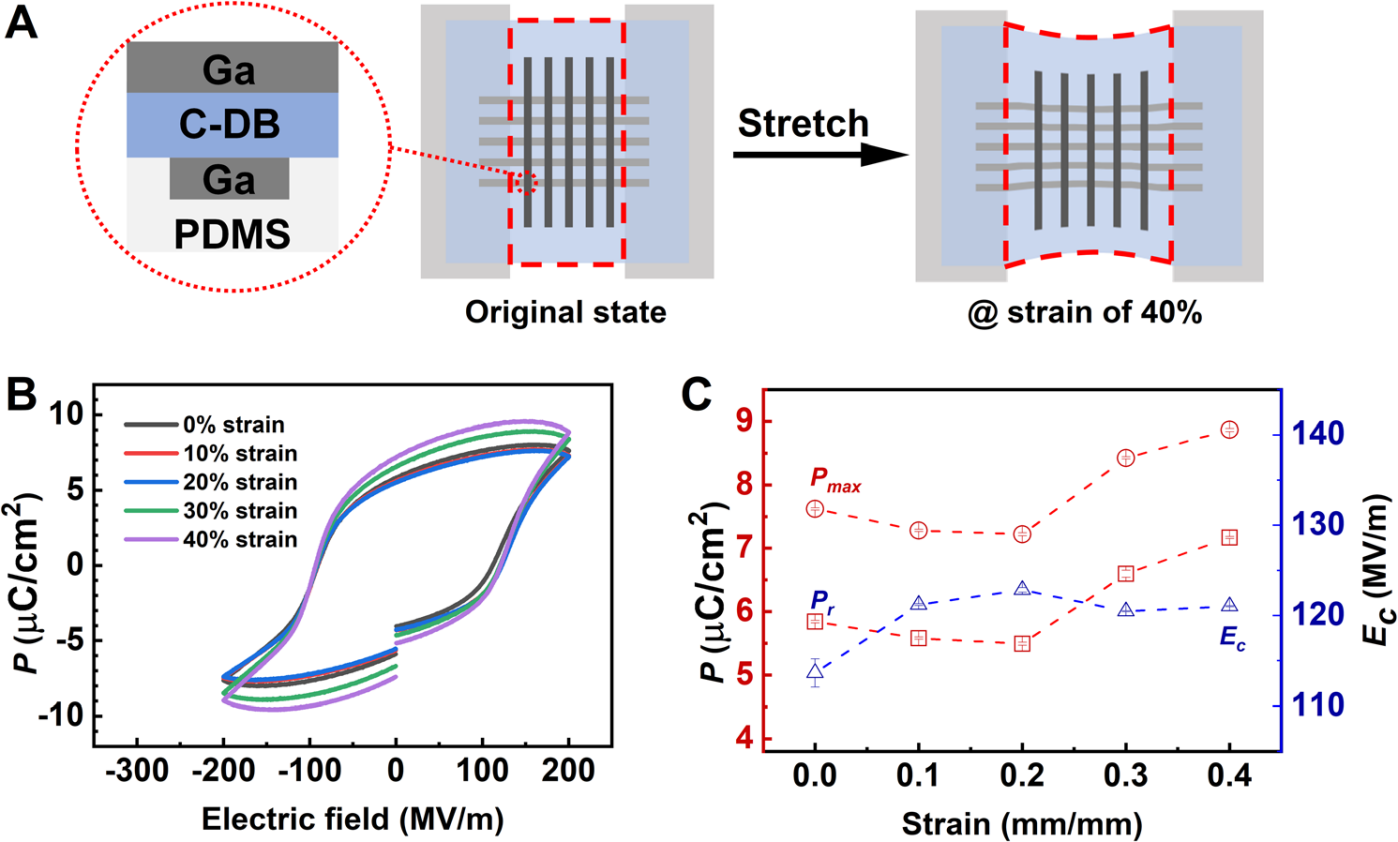
Ferroelectric response of the elastic films under strain. (A) Schematic structure of the elastic device in the stretching setup under 0–40% strain. (B) *P*–*E* loops at 1 kHz under 0–40% strain. (C) *P*_r_, *P*_max_, and *E*_c_ under different strains.

To investigate the ferroelectric response of crosslinked P(VDF-TrFE-DB) under mechanical deformation, a fully elastic capacitor device was fabricated using a sacrificial layer method with liquid metal (gallium, Ga) as the elastic electrode. The schematic of the device structure is shown in Fig. 6A. The crosslinked P(VDF-TrFE-DB) in this work has a superior modulus and crystallinity compared to earlier DB materials; however, these properties posed challenges in measuring *P*–*E* loops at high electric fields and large strains. Consequently, a maximum electric field of 200 MV m^−1^ was applied for the characterization.

The device was mounted on a custom tensile clamp and stretched incrementally to a strain of 40%. When unstrained, the *P*–*E* loops of the elastic device exhibited less rectangular hysteresis but a higher maximum polarization (*P*_max_) compared to a rigid device with Au electrodes (Fig. 6C and S13A†). Frequency-dependent *P*–*E* loops for the elastic device (Fig. S13B†) showed behavior similar to the rigid device. These observations confirmed that the ferroelectric response of the crosslinked polymer in the elastic device matched that of the rigid device under similar conditions.

To further explore the strain-dependent behavior, the device was stretched to strains ranging from 10% to 40%, and the *P*–*E* loops were recorded. With increasing strain, the loops exhibited improved rectangularity (Fig. 6B and S14–S17†), reflecting improved domain switching. Additionally, the *P*_r_, *P*_max_, and *E*_c_ values derived from the *P*−*E* loops showed a slight fluctuation with increasing strain, as presented in Fig. 6C. These results indicate that the ferroelectric response of the elastic ferroelectric materials remains stable and even slightly improves under applied strains, highlighting its potential for stretchable electronic applications.

## Conclusions

This study presents a breakthrough in designing intrinsically elastic ferroelectric polymers that achieve a high Curie temperature and significant polarization without compromising elasticity. By incorporating unsaturated bonds into commercial P(VDF-TrFE) 80/20 mol% and employing a two-step LT–HT thermal process, we successfully elevated the Curie temperature to 137 °C and achieved a remnant polarization of 7.63 μC cm^−2^, setting new benchmarks for elastic polymer ferroelectrics. The LT–HT process ingeniously balances crystallinity and amorphous regions, enabling exceptional elastic recovery with minimal crosslinker content. The high Curie temperature and large polarization not only improve operational stability but also expand the application range to harsh environments such as aerospace, automotive, and industrial systems. Furthermore, the robust ferroelectric properties and elasticity make the material promising for integration into sensors, actuators, and energy harvesting devices.

In future work, we aim to explore the integration of our materials into functional devices, particularly focusing on soft robotics and biomechanical energy conversion systems. We are also interested in tuning the polymer microstructure and crosslinking architecture to further extend the operational temperature window and optimize electromechanical coupling. These efforts will pave the way toward the practical deployment of high-performance elastic ferroelectrics in multifunctional and harsh-environment applications.

## Author contributions

Linping Wang: experimental design, experimental operation, data analysis, writing, and funding acquisition. Ben-Lin Hu: conception, experimental design, data analysis, funding acquisition, manuscript review, resources and supervision. Liang Gao, Xiaocui Rao, Fangzhou Li, Da Zu, and Yunya Liu: discussion of results and manuscript revision. All authors have given approval to the final version of the manuscript.

## Conflicts of interest

There are no conflicts to declare.

## Supplementary Material

SC-016-D5SC01467K-s001

## Data Availability

The data supporting this article have been included as part of the ESI.†
