# Supplementary material for: High-performance elastic ferroelectrics via low-temperature carbene crosslinking and high-temperature annealing
Source: Chem Sci. 2025 Apr 29;16(23):10307–14. doi: 10.1039/d5sc01467k (PMC12076213; doi:10.1039/d5sc01467k)
Supplement: SC-016-D5SC01467K-s001 [file SC-016-D5SC01467K-s001.pdf]

Supplementary Information (SI) for Chemical Science.
This journal is © The Royal Society of Chemistry 2025

Supporting Information

High-Performance Elastic Ferroelectrics via Low-Temperature Carbene Crosslinking and High-Temperature Annealing

Linping Wang, Liang Gao, Xiaocui Rao, Fangzhou Li, Da Zu, Yunya Liu, Ben-Lin Hu*

Experimental sections

Synthesis of P(VDF-TrFE-DB). The synthesis of P(VDF-TrFE-DB) was adapted from our previously established procedure.[31] Commercial P(VDF-TrFE) 80/20 mol% was sourced from Piezotech Arkema. Sodium hydroxide and acetone were supplied by National Medicines Group Chemical Reagent Co., Ltd., while hexane and *N*,*N'*-dimethylformamide (DMF) were obtained from Aladdin. In a 250 mL round-bottom flask, 5.00 g of P(VDF-TrFE) 80/20 mol% and 100 mL of DMF were added, and the mixture was stirred at 70 °C until the polymer was fully dissolved. Separately, 1.25 g of solid NaOH was dissolved in 5 mL of deionized water and further diluted with 25 mL of DMF. This diluted NaOH solution was gradually added dropwise to the P(VDF-TrFE) solution under continuous heating at 70 °C for 2 hours. At the end of the reaction, the mixture was cooled to room temperature to terminate the process. The reaction product was precipitated in deionized water, forming crude solids. These solids were then redissolved in acetone and reprecipitated in hexane. To remove residual impurities, the product was washed three times with hexane. Finally, the purified product was dried overnight in a vacuum oven at 60 °C, yielding brownish solid agglomerates.

Synthesis of crosslinking agent. The synthesis of the crosslinking agent followed the detailed procedure described in our previous work.[15]

Thick film preparation. A solution containing 100 mg of P(VDF-TrFE-DB) and varying amounts of *bis*-diazirine crosslinker was prepared in 1 mL of cyclohexanone using a vortex oscillator to ensure homogeneous mixing. The resulting solution was drop-cast onto an octadecyltrichlorosilane (OTS)-modified glass substrate in a fume hood and allowed to dry naturally for 12 hours. After most of the solvent had volatilized, the film was further dried in a vacuum oven at 60 °C for 8 hours to remove any remaining solvent. For the LT-HT (low-temperature-high-temperature) processing, the samples were crosslinked at 100 °C in a vacuum oven for 24 hours and subsequently annealed at 140 °C in a vacuum oven for 8 hours. For the HT (high-temperature) processing, the samples were crosslinked and annealed directly at 140 °C in a vacuum oven for 8 hours. The resulting thick films were then characterized by various

techniques, including differential scanning calorimetry (DSC), dynamic mechanical analysis (DMA), Fourier-transform infrared spectroscopy (FTIR), X-ray diffraction (XRD), and gel content measurement, to evaluate their structural and mechanical properties.

Thin-Film Preparation. In an Ar-charged glove box, a solution containing 50 mg of P(VDF-TrFE-DB) and 15 mg of bis-diazirine crosslinker was prepared in 0.5 mL of *N,N'*-dimethylformamide (DMF). This solution was then dropped onto an Au/Si wafer (10 mm × 10 mm), followed by spin-coating at 600 rpm for 10 seconds and 2500 rpm for 35 seconds to create a uniform film. The film was left to dry naturally in the glove box for 4 hours and then placed on a hotplate at 60 °C for 12 hours to remove any residual solvent. The dried thin film was thermally crosslinked at 100 °C for 24 hours, followed by annealing at 140 °C for 8 hours. This process resulted in a film with an approximate thickness of 600 nm. Au electrodes with a diameter of 200 μm and a thickness of 100 nm were then grown by magnetron sputtering, forming an Au/crosslinked P(VDF-TrFE-DB)/Au/Si structure. A control sample of commercial P(VDF-TrFE) thin films for rigid devices (Au/P(VDF-TrFE-DB)/Au/Si) was also prepared using the same method. The resulting thin films were used for $P{-}E$ hysteresis loop tests, atomic force microscopy (AFM) and piezoelectric force microscopy (PFM) characterizations.

Preparation of fully elastic device Measurements. The Si/SiO2 wafer was initially cleaned using oxygen plasma treatment (150 W, 3 minutes). A dextran solution (10 wt% in water) was then spin-coated onto the wafer at 600 rpm for 10 seconds and at 1500 rpm for 40 seconds. The wafer was subsequently baked on a hot plate at 80 °C for 10 minutes and at 180 °C for 30 minutes to remove any trapped water. In an Ar-charged glove box, a solution containing 50 mg of P(VDF-TrFE-DB) and 15 mg of diazirine crosslinker in 0.5 mL of *N,N'*-dimethylformamide (DMF) was prepared. The solution was spin-coated on top of the glucan layer at 600 rpm for 10 seconds and at 2500 rpm for 35 seconds. The sample was then heated at 60 °C for 12 hours to remove the solvent, followed by thermal crosslinking at 100 °C for 24 hours and annealing at 140 °C for 8 hours, all conducted within the Ar-charged glove box. After cooling to room temperature, liquid metal (LM, gallium (Ga)) was stencil-printed onto the surface of the crosslinked P(VDF-TrFE-DB) film to form strip electrode patterns. These electrodes were then encapsulated by Sylgard®™ 184 Silicone Elastomer (PDMS), with the silicone elastomer base and curing agent mixed at a ratio of 10:1, applied by spin-coating at 1500 rpm for 35 seconds. The film was cured at 60 °C overnight, resulting in a thickness of approximately 100 μm. Next, the resulting film was placed in deionized water for 1 hour to dissolve the glucan layer, after which it was peeled off from the Si/SiO2 substrate. A layer of LM (Ga) was then brushed in a

direction perpendicular to the initial LM (Ga) electrode, forming a Ga/crosslinked P(VDF-TrFE-DB)/Ga/PDMS sandwich structure. This completed the fabrication of the fully elastic capacitive array.

Calculation of DB contents. The content of VDF can be represented as the sum of the unreacted VDF content "x/2", the content of -CF=CH- bonds "y", and the content of $-CF_2-CH=CH-CF_2-$ bonds "z/2", i.e., (x/2 + y + z/2). Similarly, the content of TrFE can be expressed as the sum of the unreacted TrFE content "1", the content of -CH=CH- bonds "z/2", and the content of -CF=CF- bonds "m", i.e., (m + 1 + z/2). This relationship, with a VDF to TrFE ratio of 80:20, calculated based on the ^1H NMR (**Figure S1**), is mathematically expressed in equation (1). The total double bonds in the reaction product can be represented as (m + z/2 + y), and the content of all chemical structures can be represented as (x/2 + 1 + y + z/2 + m). Therefore, the double bond content can be expressed by equation (2). By combining equations (1) and (2), the total double bond content in the product can be calculated by equations (3).

$$\frac{20}{80}=\frac{m+1+\frac{z}{2}}{\frac{x}{2}+y+\frac{z}{2}}\# (1)$$

$$m=\frac{x+2y-3z-8}{8}\# (2)$$

$$Content(DB)=\frac{m+y+\frac{z}{2}}{\frac{x}{2}+1+m+y+\frac{z}{2}}=\frac{x+10y+z-8}{5x+10y+z}\# (3)$$

Here, $x=\int_{2.30}^{3.30}VDF$, $y=\int_{6.20}^{6.36}CFCH$, and $z=\int_{6.72}^{6.86}CHCH$ from ^1H NMR integral. The contents of "$-CF_2-CH=CH-CF_2-$", "-CF=CH-", and "-CF=CF-" were determined to be 0.3 mol%, 0.4 mol%, and 1.1 mol%, respectively, giving a total DB content of 1.8 mol%.

Calculation of "head to tail structure" content. The regiosequences in the ^{19}F NMR spectra were assigned according to the method described in previous works as shown in **Figure S2** and **Table S1&2**.

Calculation of crosslinking density. The crosslinking density (ρ) was defined as the fraction of the cross-linking points (v) in the total repeat units (N_0), represented by the equation $\rho = v/N_0$. For the crosslinked P(VDF-TrFE-DB) films, the crosslinking points were calculated to be twice

the amount of the *bis*-diazirine crosslinker. The average molecular weight of the repeat unit in P(VDF-TrFE-DB) is 67.49 (calculated as $0.796 \times 64.03 + 0.186 \times 82.03 + 0.003 \times 126.05 + 0.004 \times 44.03 + 0.011 \times 62.02 = 67.49$, where the molecular weights of VDF, TrFE and DBs are 64.03, 82.03, 126.05, 44.03, and 62.02, respectively). Therefore, the crosslinking density (ρ) in the cross-linked P(VDF-TrFE-DB) can be calculated using equation (4):

$$\rho = 2(a/2765.17)/(b/67.49) \quad (4)$$

where a/b is the weight ratio of *bis*-diazirine crosslinker to P(VDF-TrFE-DB), and 2765.17 is the molecular weight of *bis*-diazirine crosslinker. The corresponding weight ratios (diazirine crosslinker to P(VDF-TrFE-DB)) and crosslinking densities used in this study are listed in **Table S4.**

Measurement methods

NMR measurement. ^{1}H NMR and ^{19}F NMR spectra were acquired using a Bruker 600 MHz spectrometer (Advance NEO 600, Switzerland). The acquisition involved 128 scans for each spectrum. The content analysis was performed with reference to previous studies.[35-37]

Strain-stress measurements. The mechanical properties of the samples were assessed through strain–stress analysis using an Instron-5943 instrument. The initial dimensions of the film were set to 10 mm in length and 3 mm in width. A stretching rate of 5 mm/min was consistently applied for all stress–strain curves. The cyclic stretching curves, as depicted in **Figure 3C**, were obtained under progressively increasing strain. The first cycle was omitted owing to clamp sliding. To enhance clarity in presenting the stretch and release loops, the curves at each strain were shifted along the x-axis. Additionally, cyclic stretching curves shown in **Figure S6** were obtained with over 7000 repetitions utilizing a Cellscale instrument (Univert S2, Canada).

DSC analysis. Differential scanning calorimetric (DSC) analysis was conducted on a DSC calorimeter (214, Netzsch, Germany) in a nitrogen atmosphere at a scanning rate of 10 °C/min during the heating and cooling cycles.

TG analysis. Thermogravimetric analysis (TGA) of the cross-linked P(VDF-TrFE-DB) was conducted by a thermogravimetric analyzer (TG209F1, Netzsch, Germany) in a nitrogen atmosphere at a scanning rate of 10 °C/min from room temperature to 800 °C.

FTIR. Micro-infrared absorption spectra of the films were recorded using a microscopic infrared spectrometer (Cary660, Agilent, USA). The number of scans was set to 32 for enhanced accuracy.

X-ray diffraction. High-resolution X-ray diffraction measurements (Theta-2theta) were conducted using an X-ray Powder diffractometer (D8 ADVANCE DAVINCI, Bruker, Germany). The X-ray wavelength utilized was 1.54 Å (Cu Kα radiation, 40 kV, and 100 mA), and the scanning rate was maintained at 4 °/min.

***P–E* hysteresis loop.** Electric P–E hysteresis loops at room temperature were acquired using a Premiere II ferroelectric tester from Radiant Technologies, Inc., USA. AC electric fields with increments of 25 and 50 MV/m were applied across the polymer films with a triangular waveform in the frequency range of 500 Hz to 10 kHz. Film thicknesses at three points were measured using a surface profiler (Dektak XT, Bruker, USA) to obtain the average thickness.

AFM characterization. AFM morphologies were characterized using a Scanning Probe Microscope (Dimension ICON, Bruker, USA). Piezoresponse Force Microscopy (PFM) measurements were performed with a commercially available piezoresponse force microscope (Oxford Instruments, Cypher ES) equipped with a high-voltage package. Diamond coated conductive tip DCP11 were used for polarization switching investigations.

Dynamic mechanical analysis. To discern the individual contributions of energy elasticity and entropy elasticity, stress-temperature relationship of crosslinked P(VDF-TrFE-DB) was obtained using a dynamic mechanical analyzer (DMAQ800, TA instruments, America) within a specific temperature range.

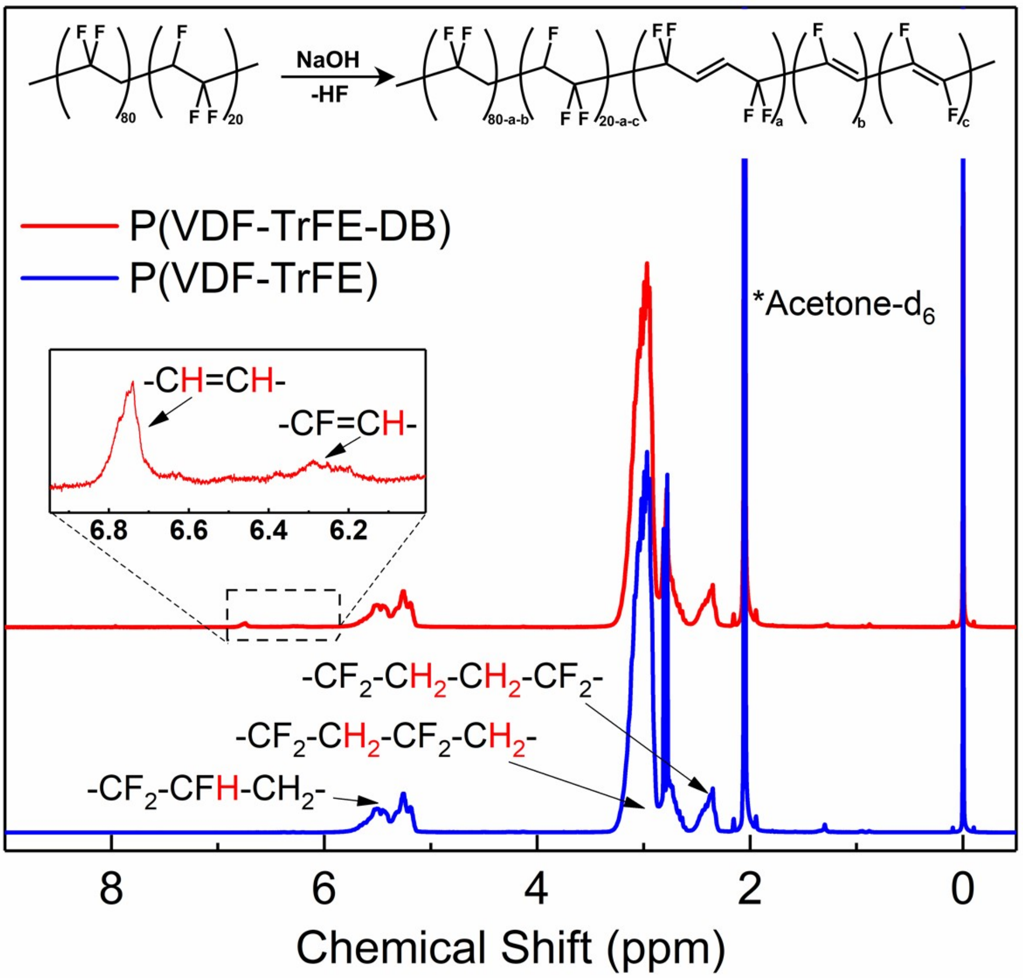

Figure S1. ^{1}H NMR spectra of P(VDF-TrFE) and P(VDF-TrFE-DB).

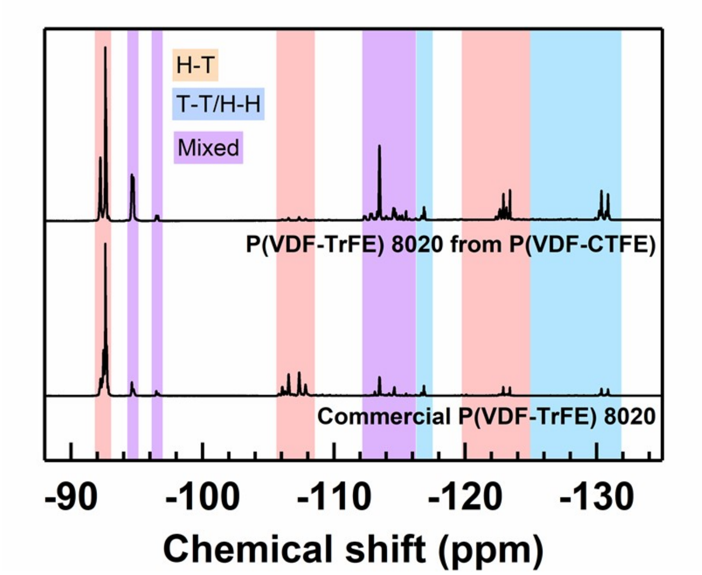

Figure S2. ^{19}F NMR spectra of P(VDF-TrFE) and P(VDF-TrFE-DB).

Table S1. Assignments of ^{19}F NMR signals of P(VDF-TrFE). The monomers indicated by the ^{19}F NMR signals are underlined. H–H, head to head; H–T, head to tail; T–T, tail to tail.

Regioregularity	5C Sequence	Designation	Chemical Shift (ppm)	Integral area of 19 F NMR for Commerc-ial 8020	Integral area of 19 F NMR for 8020 from P(VDF-CTFE)
H-T or T-H	CF2CH2**CF2**CH2CF2	**VDF-VDF, H-T**	**-93.2**	**1**	**1**
	CF2CH2**CF2**CHFCF2	**VDF**-TrFE, H-T	-107.8	0.408	0.230
	CF2CHF**CF2**CHFCF2	**TrFE**-TrFE, H-T	-119.5 to -124.8	0.089	0.101
	CHFCF2**CHF**CF2CHF	**TrFE**-TrFE, T-H	-207.3 to -213.5	/	/
	CH2CF2**CHF**CF2CH2	**TrFE**-VDF, T-H	-197.5 to -201.5	/	/
H-H/T-T or T-T/H-H	CH2CH2**CF2**CF2CH2	VDF-**VDF**-VDF, T-T/H-H	-117.2	0.051	0.013
	CHFCHF**CF2**CF2CHF	TrFE-**TrFE**-TrFE, T/H-H	-124.8 to -130.0	0.062	0.078
	CH2CHF**CF2**CF2CH2	VDF-**TrFE**-VDF, T/H-H-H	-131.1	/	/
	CF2CF2**CHF**CHFCF2	VDF-**TrFE**-TrFE, H-H/T-T	-218.5 to -220.4	/	/
Others	CHFCH2**CF2**CH2CF2	TrFE-**VDF**-VDF, T/T-H/T	-94.8 to -95.8	0.086	0.305
	CH2CH2**CF2**CH2CF2	VDF-**VDF**-VDF, T/T-H-T	-96.3 to -97.8	0.027	0.067
	CF2CH2**CF2**CF2CHF	VDF-**VDF**-TrFE, T/H-H-H	-114.2	0.184	0.384

Table S2. H-T, H–H/T-T, and mixed structures of two types of P(VDF-TrFE)

	H-T normal structures	H-H/T-T regiodefects	Others
Commercial 8020	78.5%	5.9%	15.6%
8020 from P(VDF-CTFE)	61.1%	4.2%	34.7%

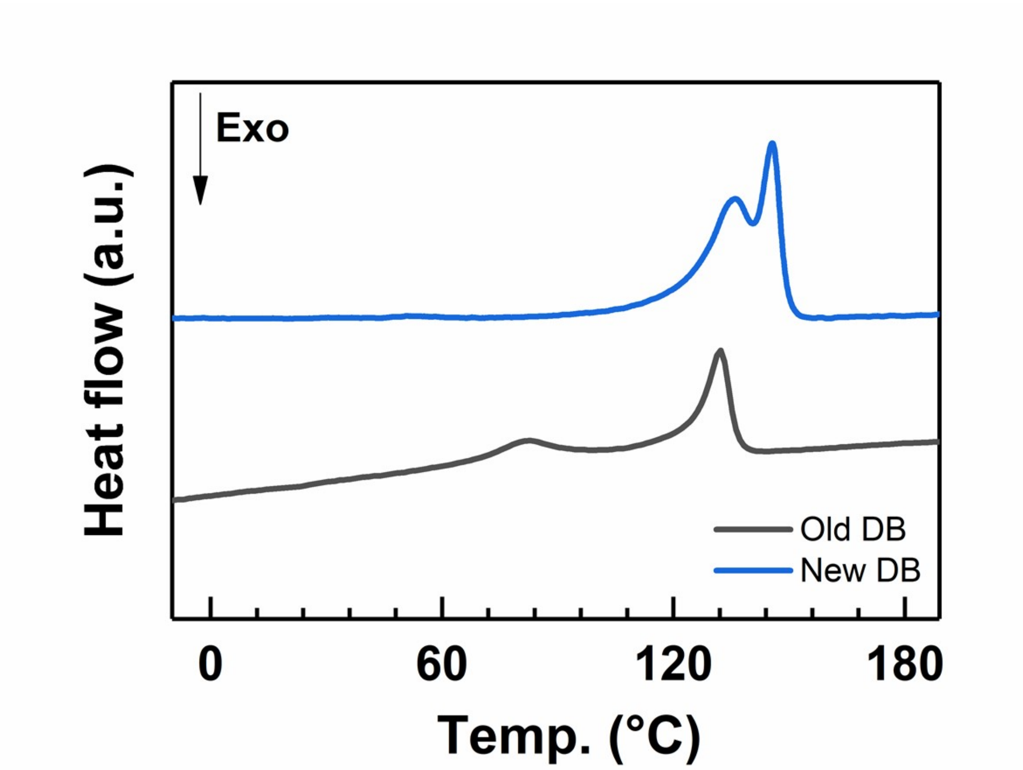

Figure. S3. DSC curves of old and new DBs

Table S3. Properties of two DBs

	T_c	T_m	ΔH_{c+m}	**Tensile Break**
DB in the previous work[14]	~82 °C	~132 °C	33 J/g	~700%
**DB in this work**	**~136 °C**	**~146 °C**	**54 J/g**	**~250%**

Table S4. Calculation of crosslinking density

Weight ratio (a/b)	0	10:100	20:100	30:100	40:100	50:100	60:100
Crosslinking density (%)	0	0.5	1.0	1.5	2.0	2.5	3.0

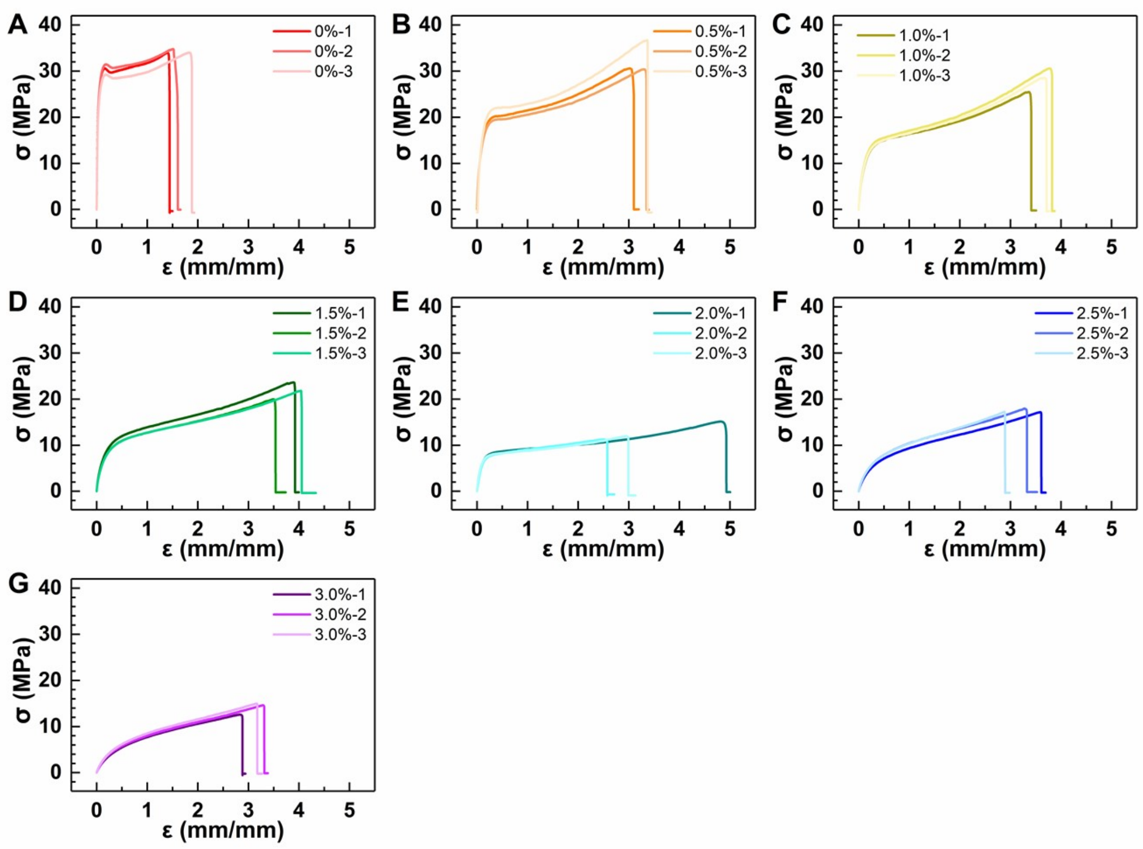

Figure S4. Stress–strain curves of crosslinked P(VDF-TrFE-DB) thick films at various crosslinking densities via LT-HT process.

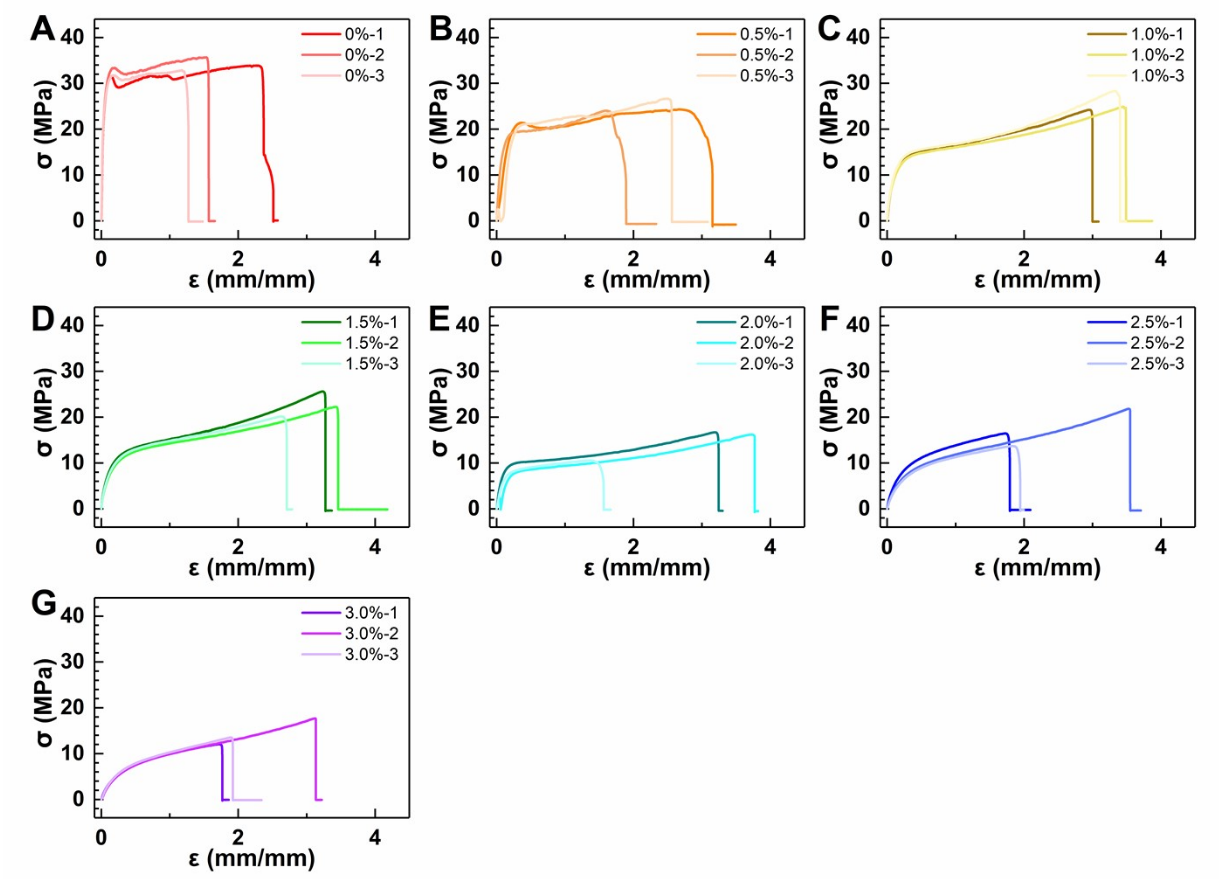

Figure S5. Stress–strain curves of crosslinked P(VDF-TrFE-DB) thick films at various crosslinking densities via HT process.

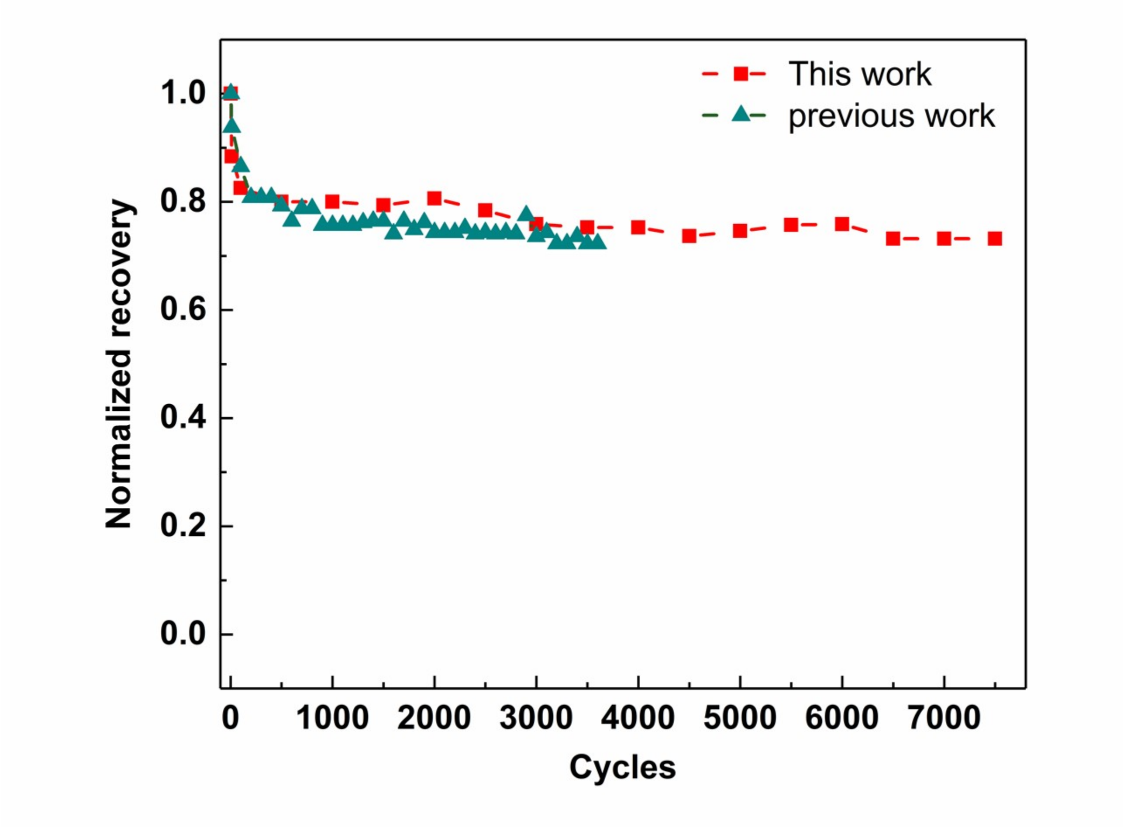

Figure S6. Fatigue performances of crosslinked P(VDF-TrFE-DB) film under cyclic strain of 50%, compared to that of old crosslinked P(VDF-TrFE-DB) film from our previous work.[14]

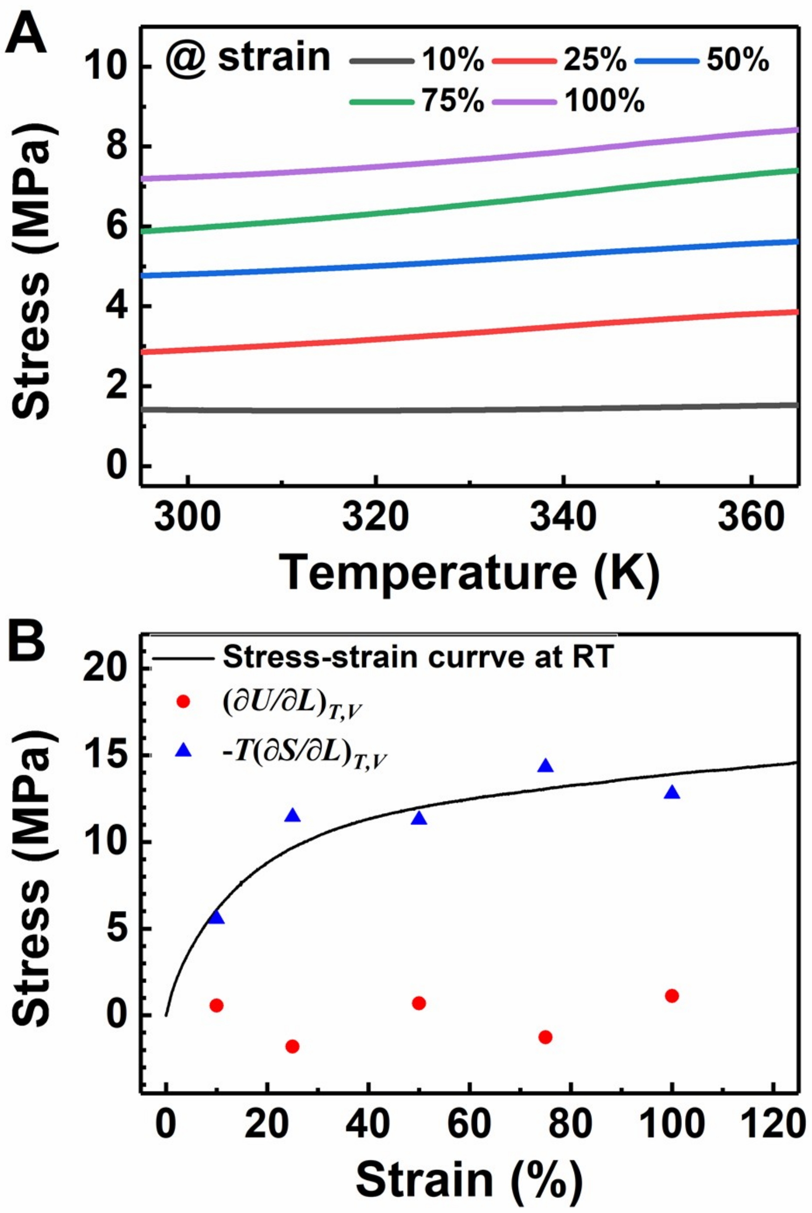

Figure S7. Contributions of energy elasticity and entropy elasticity in crosslinked P(VDF-TrFE-DB). (A) Force-temperature relationships (*f*–*T* curves) of crosslinked P(VDF-TrFE-DB) film. (B) Internal energy and entropy components of tensile stress as functions of strain for crosslinked P(VDF-TrFE-DB) film, compared to the stress–strain curves at 25 °C.

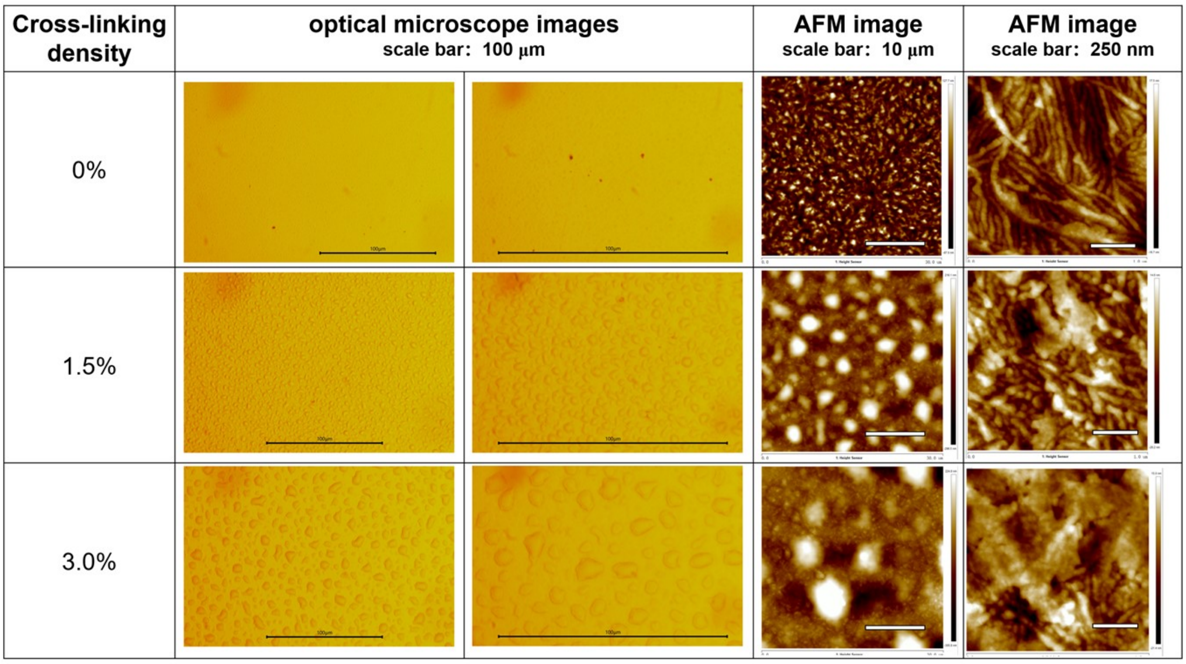

Figure S8. Optical microscopy and AFM height images of pristine and crosslinked P(VDF-TrFE-DB) thin films at different crosslinking densities.

Table S5. Enthalpy change and crystallinity of the pristine and crosslinked P(VDF-TrFE-DB) during Curie transition and melting process of VDF

Crosslinking density (%)	0%	0.5%	1.0%	1.5%	2.0%	2.5%	3.0%
ΔH_{PEG} (J/g)	0	low	low	low	2.8	5.7	10.0
ΔH_{c+m} (J/g)	54	46	44	43	38	36	34
ΔX_c of VDF	69.0%	64.6%	67.4%	71.4%	67.9%	69.0%	69.5%

The enthalpy changes are represented by ΔH_{PEG} for the melting process of PEG chains, and ΔH_{c+m} for the Curie transition and melting processes of both pristine and crosslinked P(VDF-TrFE-DB). The degree of crystallinity is denoted by ΔX_c. For VDF, the formula is $\Delta X_c = \Delta H_{c+m}/(a*\omega(VDF)*\Delta H_0)$, where "a" is the mass ratio of P(VDF-TrFE-DB) in the crosslinked films, ω(VDF) is the mass ratio of VDF in the P(VDF-TrFE-DB) copolymers (75.74%), and ΔH_0 of VDF is the melting enthalpy of 100% crystalline VDF (103.4 J/g).[35]

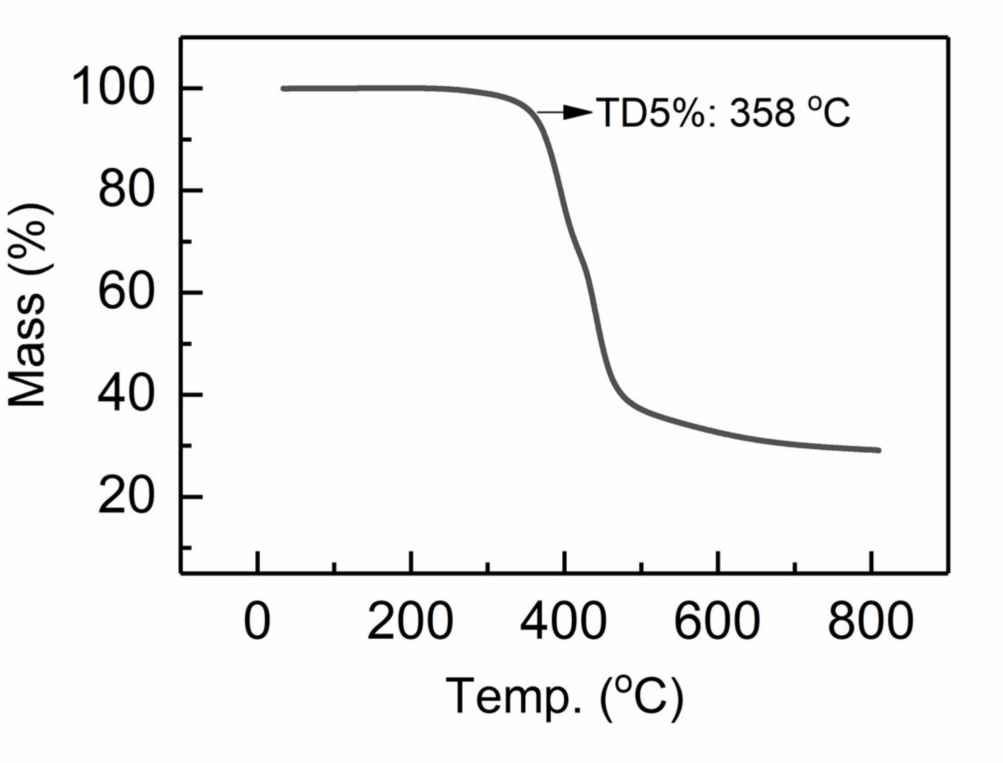

Figure S9. Thermogravimetric diagram of the crosslinked P(VDF-TrFE-DB) film with a crosslinking density of 3%.

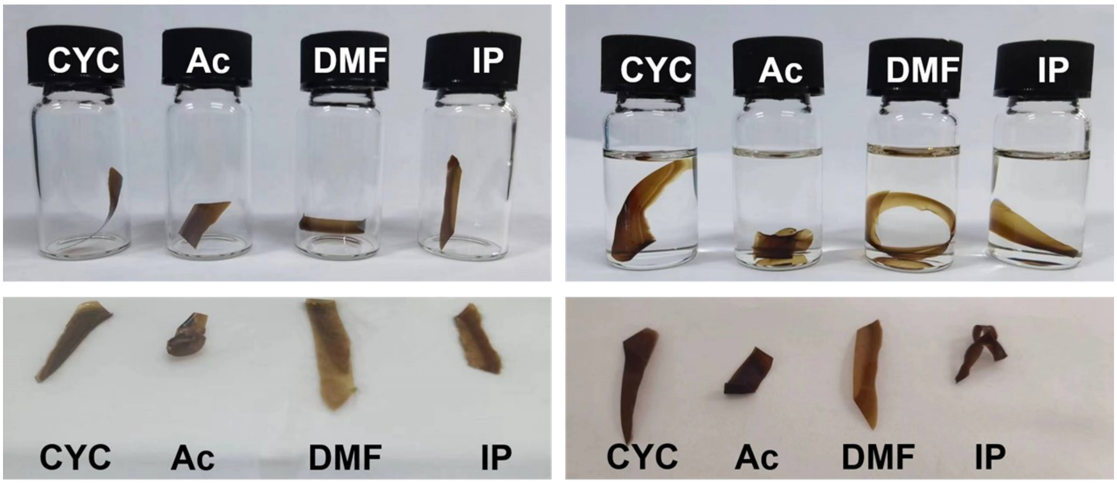

Figure S10. Photos of crosslinked P(VDF-TrFE-DB) films immersed in different solvents: (A) after 10 min; (B) after 1 day; (C) after 12 days; (D) after drying.

Table S6 Gel content of crosslinked P(VDF-TrFE-DB) immersed in different solvents

Solvent	Original mass /mg	Final mass /mg	Ratio of gel
CYC	9.47	9.33	98.5%
Ac	8.14	7.50	92.1%
DMF	8.13	7.57	93.1%
IP	7.66	6.29	82.1%

CYC: cyclohexanone; Ac: acetone; DMF: *N,N*-dimethylformamide; IP: isophorone.

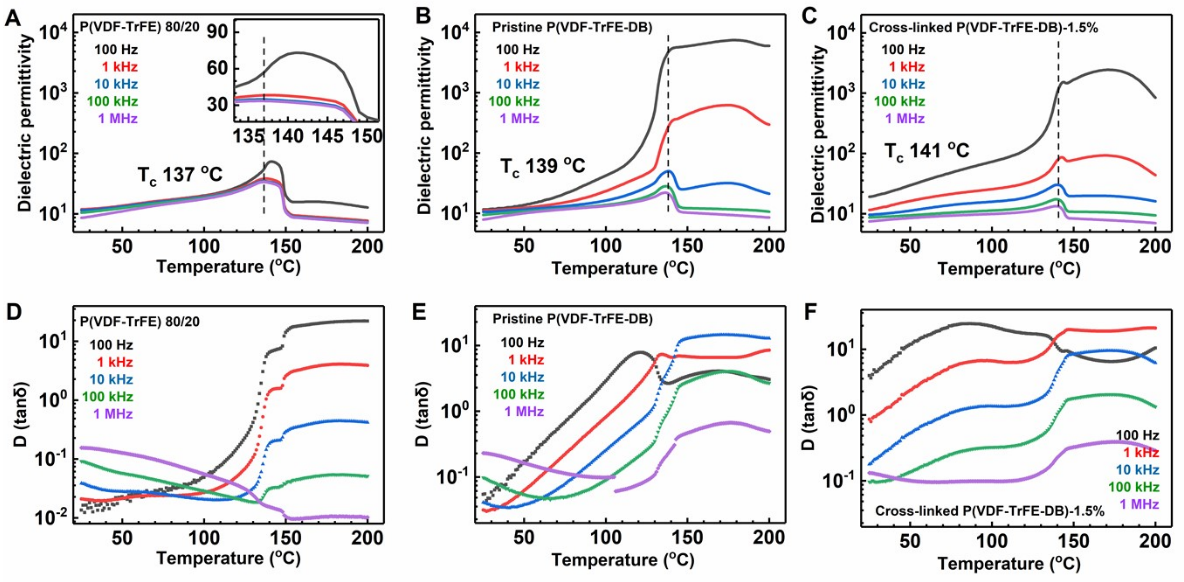

Figure S11. Temperature-dependent (ε-T) curves of pristine P(VDF-TrFE-DB) and crosslinked P(VDF-TrFE-DB) films at selected frequencies. Dielectric permittivity of (A) pristine P(VDF-TrFE-DB) and (B) crosslinked P(VDF-TrFE-DB); Dielectric loss of (C) pristine P(VDF-TrFE-DB) and (D) crosslinked P(VDF-TrFE-DB).

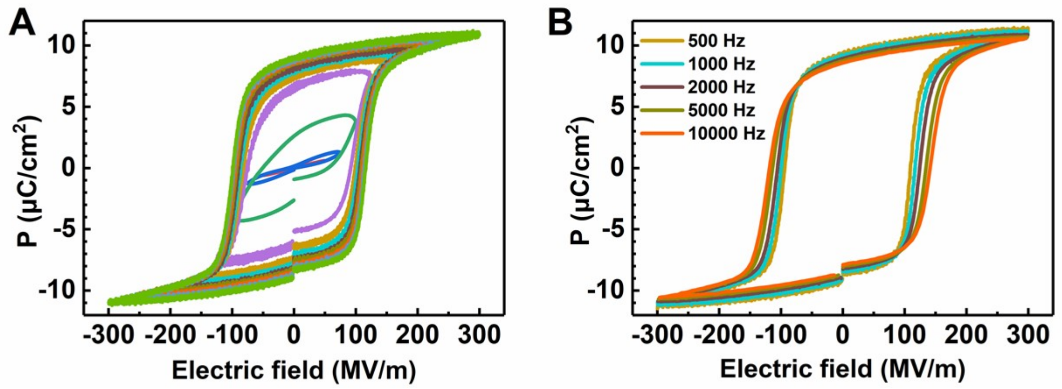

Figure S12. $P-E$ loops of commercial P(VDF-TrFE) (A) under increased electric fields at 1000 Hz and (B) with increased frequencies.

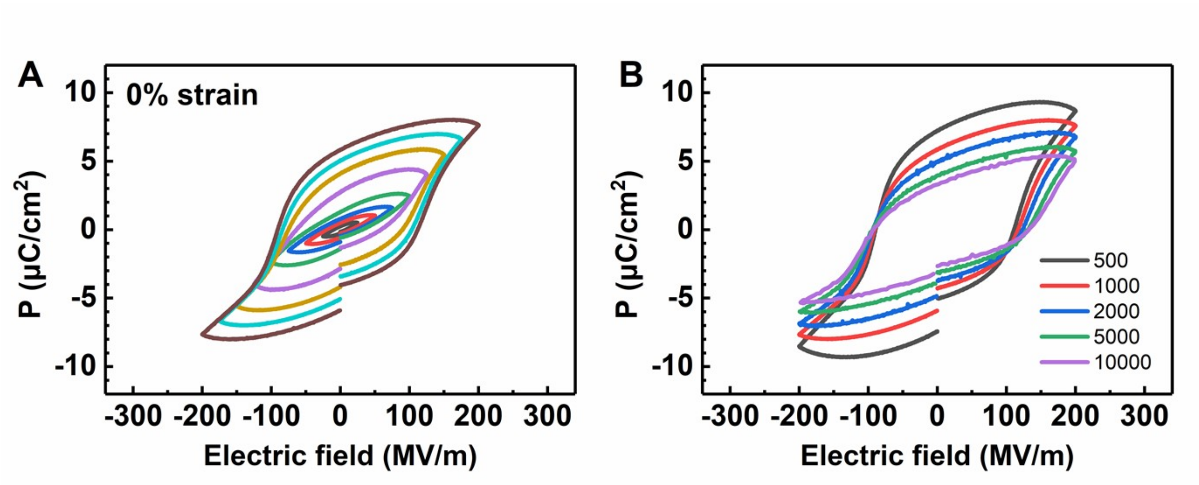

Figure S13. $P\!-\!E$ hysteresis loops of Ga/crosslinked P(VDF-TrFE-DB)/Ga/PDMS without stress (A) at 1000 Hz under various electric fields and (B) at various frequencies under 200 MV/m.

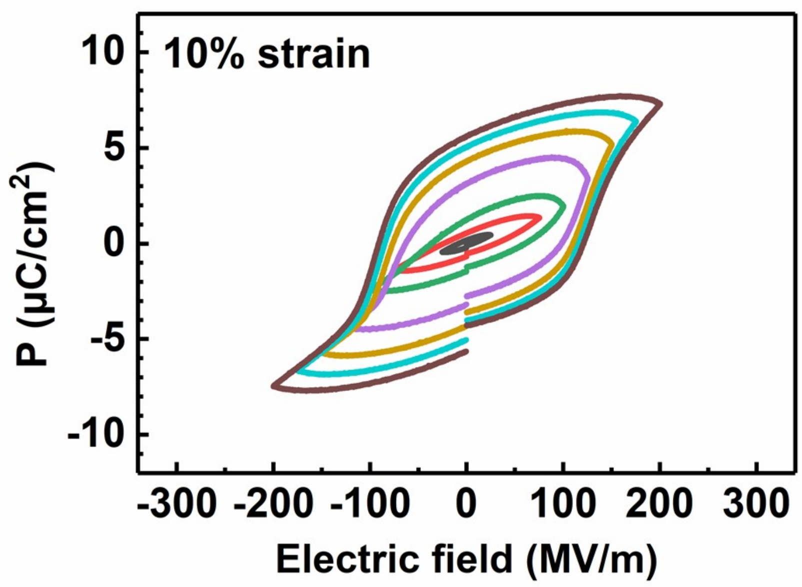

Figure S14. $P-E$ hysteresis loops of Ga/crosslinked P(VDF-TrFE-DB)/Ga/PDMS under 10% tensile strain at 1000 Hz.

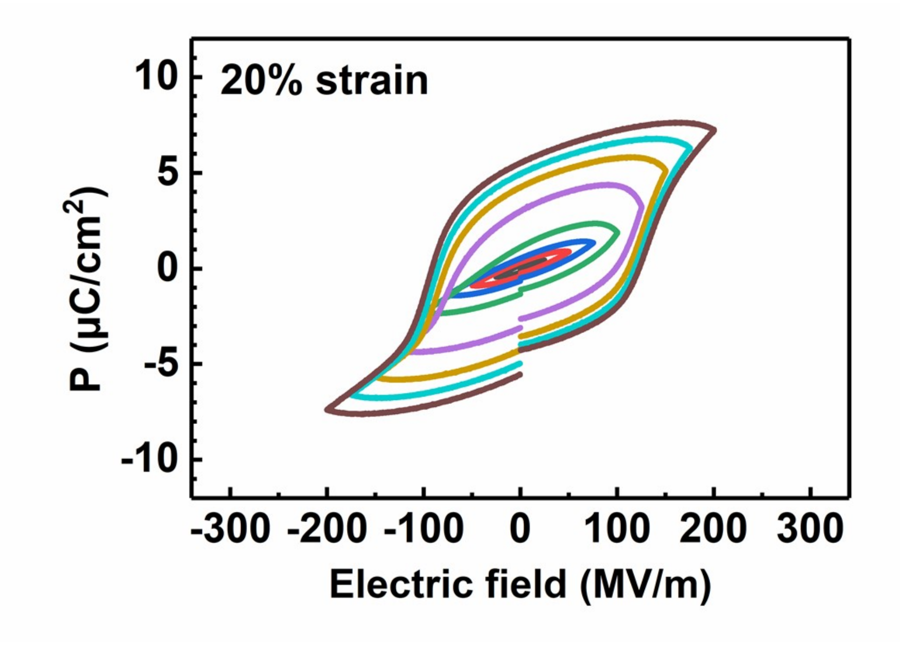

Figure S15. *P−E* hysteresis loops of Ga/crosslinked P(VDF-TrFE-DB)/Ga/PDMS under 20% tensile strain at 1000 Hz.

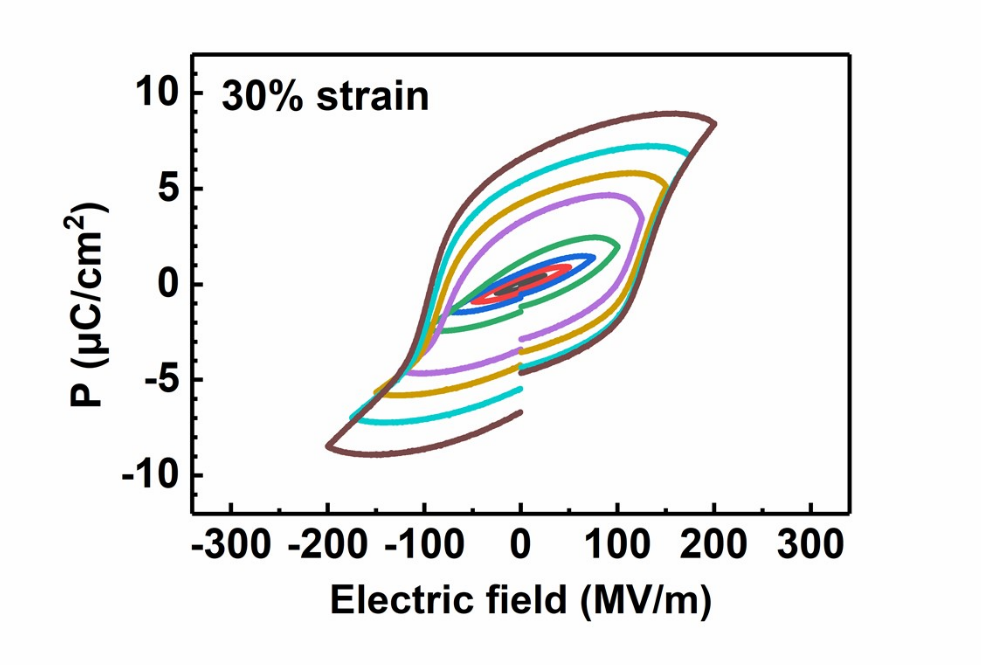

Figure S16. *P−E* hysteresis loops of Ga/crosslinked P(VDF-TrFE-DB)/Ga/PDMS under 30% tensile strain at 1000 Hz.

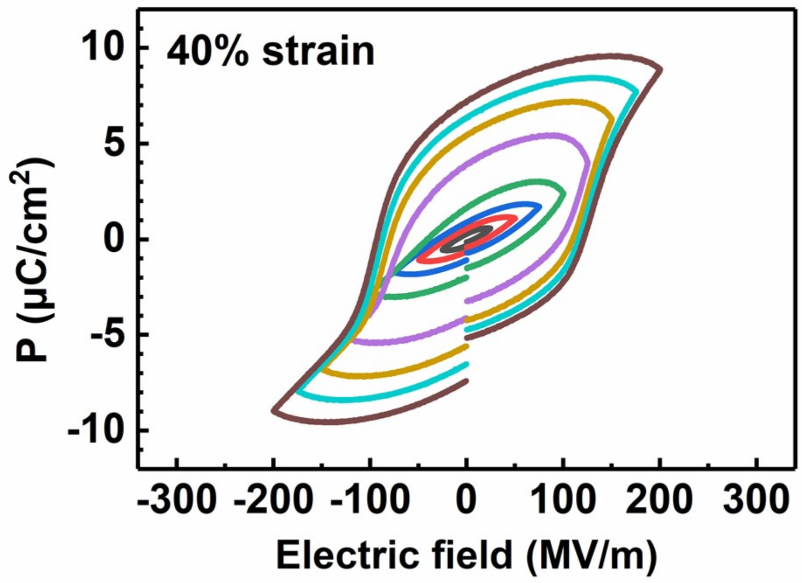

Figure S17. $P{-}E$ hysteresis loops of Ga/crosslinked P(VDF-TrFE-DB)/Ga/PDMS under 40% tensile strain at 1000 Hz.
